# The Bovine Pangenome Consortium: democratizing production and accessibility of genome assemblies for global cattle breeds and other bovine species

**DOI:** 10.1186/s13059-023-02975-0

**Published:** 2023-06-19

**Authors:** Timothy P. L. Smith, Derek M. Bickhart, Didier Boichard, Amanda J. Chamberlain, Appolinaire Djikeng, Yu Jiang, Wai Y. Low, Hubert Pausch, Sebastian Demyda-Peyrás, James Prendergast, Robert D. Schnabel, Benjamin D. Rosen, Bovine Pangenome Consortium

**Affiliations:** 1 US Meat Animal Research Center, USDA-ARS, Clay Center, NE 68933 USA; 2 Dairy Forage Research Center, USDA-ARS, Madison, WI 53706 USA; 3 Université Paris-Saclay, INRAE, AgroParisTech, GABI, 78350 Jouy-en-Josas, France; 4 Agriculture Victoria, AgriBio, Centre for AgriBioscience, Bundoora, VIC 3083 Australia; 5 School of Applied Systems Biology, La Trobe University, Bundoora, VIC 3083 Australia; 6 Centre for Tropical Livestock Genetics and Health, ILRI Kenya, Nairobi, 30709-00100 Kenya; 7 Centre for Tropical Livestock Genetics and Health, Easter Bush, Midlothian, EH25 9RG UK; 8 Center for Ruminant Genetics and Evolution, Northwest A&F University, Yangling, 712100 China; 9 The Davies Research Centre, School of Animal and Veterinary Sciences, University of Adelaide, Roseworthy, SA 5371 Australia; 10 Animal Genomics, ETH Zurich, Universitaetstrasse 2, 8092 Zurich, Switzerland; 11 Departamento de Producción Animal, Facultad de Ciencias Veterinarias, Universidad Nacional de La Plata, 1900 La Plata, Argentina; 12 Consejo Superior de Investigaciones Científicas Y Tecnológicas (CONICET), CCT-La Plata, 1900 La Plata, Argentina; 13 The Roslin Institute, University of Edinburgh, Easter Bush, Midlothian, EH25 9RG UK; 14 Division of Animal Sciences, University of Missouri, Columbia, MO 65211 USA; 15 Animal Genomics and Improvement Laboratory, USDA-ARS, Beltsville, MD 20705 USA

## Abstract

The Bovine Pangenome Consortium (BPC) is an international collaboration dedicated to the assembly of cattle genomes to develop a more complete representation of cattle genomic diversity. The goal of the BPC is to provide genome assemblies and a community-agreed pangenome representation to replace breed-specific reference assemblies for cattle genomics. The BPC invites partners sharing our vision to participate in the production of these assemblies and the development of a common, community-approved, pangenome reference as a public resource for the research community (https://bovinepangenome.github.io/). This community-driven resource will provide the context for comparison between studies and the future foundation for cattle genomic selection.

**Supplementary Information:**

The online version contains supplementary material available at 10.1186/s13059-023-02975-0.

## Why a pangenome?

The inadequacy of a single reference assembly for the detection of all genetic variation has been documented with respect to human genome research (reviewed in [1] and [2]). A broad consensus of the biomedical genomics research community supports the creation of highly accurate and haplotype-phased assemblies that include examples from across common human haplotypes, followed by the construction of a pangenome representation to underpin variant detection. Existing variation detection tools have been reported to be sensitive to the quality and representation of the reference genome (i.e., reference bias) and to be improved by the use of more representative reference(s) or pangenome representations [3]. Structural variants are particularly difficult to accurately identify, characterize, and compare using now-standard approaches of short-read or even long-read mapping to a single-haplotype reference assembly [4]. Insertions relative to the reference sequence are particularly recalcitrant to detection without orthogonal genomic information as sequence reads from haplotypes containing the insertion fail to properly map to the reference. Sequence reads with low similarity to the reference assembly due to population variation in genome sequence can cause incorrect mapping and lead to incorrect genotypes in whole-genome shotgun (WGS)-based genotyping assays. One example of reference bias resulting in genotype errors is the human leukocyte antigen locus, where data from the 1000 Genomes Project suggested that nearly 19% of SNPs were incorrectly identified [5]. As many as 68% of structural variants detected by alignments of haplotype-resolved assemblies were not detected using short-read mapping to the human reference assembly [6], highlighting the need for a more complete reference genome resource for such analyses. The development of a human pangenome through the Human Pangenome Reference Consortium (HPRC) has been implemented (https://humanpangeome.org) by way of a Human Pangenome Reference Sequence Project, funded with nearly US $30 million in grants in 2019 (https://www.genome.gov/news/news-release/NIH-funds-centers-for-advancing-sequence-of-human-genome-reference). The charter for this effort includes the ethical access of samples to avoid the exploitation of local populations and outreach to international groups as explicit goals [7]. Furthermore, the HPRC project seeks to address limitations inherent in contemporary genetic variant surveys using individual, linear genome assemblies, and computational tools designed for comparisons against a lone reference [7]. Because no similar singular funding source is available for agricultural genomics, the Bovine Pangenome Consortium (BPC) has been launched (https://bovinepangenome.github.io/) to coordinate distributed efforts within the global bovine genomics community towards achieving similar ends.

The goal of genomics research in livestock has been to correlate variation in genome sequence with phenotypes that affect traits of importance to animal health, welfare, productivity, profitability, and sustainability as part of a broader strategy to increase favorable allele frequency and decrease deleterious allele prevalence. The importance of this research is highlighted by the recent investment of up to US $40 million per year in the “Agricultural Genomes to Phenomes Initiative” by the USDA [8] and the establishment of the international Functional Annotation of Animal Genomes “FAANG to Fork” initiative [9], a project analogous to the human ENCODE project [10]. Prior work in the development of predictive models for complex trait phenotypes from a fixed set of genetic markers, termed genomic selection, has resulted in substantial benefits for animal breeders [11, 12]. Implementation of genomic selection in dairy cattle has doubled the rate of economic gain transmitted by Holstein bulls in the US Dairy Industry, resulting in profits for breeders and more efficient milk production within the system [13]. However, the accuracy of genomic selection on certain phenotypic traits is greatly impacted by environmental effects and the influence of high-effect, low-frequency genetic variants [14]. Improved reference genome assemblies and efforts to predict combined annotation-dependent depletion scores (CADD; [15]) for all bases in the genome of a species could allow for the inclusion of high-effect, low-frequency variants into generalized genomic selection models. The entire economic impact of the US Dairy Industry alone has been estimated to be $753 billion (USD), which makes even small improvements in system efficiency valuable in terms of nominal economic value (https://www.idfa.org/dairydelivers). For example, a 1% increase in the reliability of predicting production traits from genotype data made possible by the implementation of an improved reference assembly in dairy breed genomic evaluation would amount to an improvement in the efficiency of $7.5 billion (USD) in the entire system [16].

The establishment of a cattle reference genome was foundational to the conduct of genetic/genomic analysis and transformational for the practice of evaluating genetic potential. The ARS-UCD1.2 reference assembly [17] has profound limitations as it is a haploid representation of a single Hereford cow selected from a line-bred herd with high historical inbreeding [18]. This reference assembly was useful but inherently limits the ability to analyze the breadth of genome variation existing in global cattle populations. We suggest the development of reference-quality genome assemblies for as many of the existing distinct cattle breeds, including representatives of both subspecies, *Bos taurus taurus* and *Bos taurus indicus*, henceforth taurine and indicine, as practical to modernize the cattle reference genome. These assemblies would then be used to create a new, globally representative reference genome graph as a resource for future genomics studies. We propose that this “pangenome” graph should focus on the careful selection of structural variant sites across cattle breeds to optimize the utility of the graph for different research purposes [3, 19, 20].

Additional members of the Bovini tribe, henceforth bovine, are also of particular interest as they represent lineages that have been subjected to both natural and artificial selection and bottlenecks throughout their history. Taurine and indicine cattle, riverine and swamp buffalo (*Bubalus bubalis*), yak (*Bos grunniens*), and gayal (*Bos frontalis*) have extensive histories of domestication which include the recent formation of breeds due to intensive selection for agricultural food products. Other bovines, such as bison (*Bison bison*), wisent (*Bison bonasus*), banteng (*Bos javanicus*), wild yak (*Bos mutus*), gaur (*Bos gaurus*), and cape buffalo (*Syncerus caffer caffer*), exist as wild populations that have experienced varying natural constraints on mating. Comparative genomic analysis of these divergent lineages requires suitable genomics resources and integration of reference genome assemblies into a bovine pangenome will facilitate the investigation of these evolutionary processes as well as increase our understanding of potential introgression events that may have occurred throughout history.

Complete functional annotation of genes, transcript isoforms, chromatin states and their variation between cell types and cell functions, and accurate mapping of three-dimensional structures of chromatin are all dependent on complete knowledge of the cattle genome including all extant haplotypes. Assessment of the value of preserving disappearing or threatened breeds would benefit from genome-level analysis documenting any unique contribution they might provide for maintaining the full extent of existing variation in the species and evaluating any unique traits they may harbor. The inclusion of breeds common in low- and middle-income countries will support the application of genomics in situations where mass genotyping of animals is not feasible. Comparison to genome assemblies of other bovines will provide important context for assessing the conservation of genomic loci that may underlie phenotypic diversity. Pangenome research that encompasses both wild and domesticated relatives of cattle can contribute to research in other food animal bovids, illuminate the genomic consequences and mechanisms of domestication, and provide insight into the genes involved with docility and other behaviors and adaptation traits. These objectives, in addition to the improvement of detection of structural variants and increased accuracy of SNP calling from WGS data, provide the primary motivation for constructing a bovine pangenome. A properly constructed and widely used pangenome should ideally replace any need for breed-specific assemblies in most genome research applications while facilitating across-study comparisons.

## Goals of a bovine pangenome project

The global cattle population appears much more diverse than the human population, with intra-individual heterozygosity up to 3- to tenfold higher in cattle [21]. The bottlenecks associated with domestication and spread of cattle populations have not reduced the effective population size of the global population to the extent observed for *Homo sapiens*. This suggests that the construction of a cattle pangenome would provide an even higher impact in the identification and evaluation of genomic variation than has been documented or is expected in biomedical research on human populations. There are > 1000 recognized breeds across the globe, representing the two subspecies, with population sizes ranging from the hundreds to tens of millions. The estimated high diversity of ancestral aurochs that formed the basis for at least two distinct domestication events has led to a relatively high degree of genetic heterogeneity even in breeds with small effective population sizes. This diversity is reflected in the success of selection for dairy traits in Holstein cattle, for example, where a very small effective population size has not interfered with substantial progress in phenotype- or genotype-based selection [22]. The plethora of breeds and high within-breed heterogeneity makes it imperative to include as many breeds as possible in the construction of a pangenome. Our ability to represent all structural variation will be a function of allele frequencies and the partitioning of variation between breeds. It will therefore be important to test the necessity of including multiple assemblies within more cosmopolitan breeds where animal numbers are high, or the breed may have had its genome shaped by different selection pressures.

**Fig. 1:**
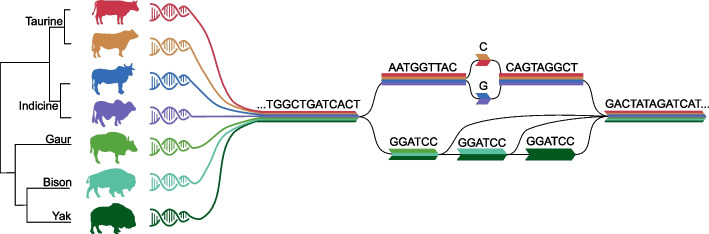
Bovine species relationships and pangenome conceptualization. Single nucleotide and structural variation between species/subspecies/breeds are depicted as different paths through a multi-species pangenome

Genome assemblies of domesticated and wild bovine relatives have value for rooting phylogeny through the identification of ancestral alleles in regions that are less conserved at a wider evolutionary scale; they provide context on the evolution of chromosomes, centromere positioning, and the origin of structural variants; they support detection and characterization of genetic variation related to domestication including docility traits that were likely the original artificial selection; they allow comparisons of the genomic sequence which may expose novel genes or incomplete lineage sorting [23] that may have influenced species/breed domestication outcomes and may be targets of future selection. For these reasons, the establishment of reference-quality genome assemblies of these species is being pursued as part of our initiative. Assemblies of bovine relatives of cattle will also be assessed for potential utility in the construction of a pangenome graph resource for cattle lineages, although the relative value of this is still under investigation (Fig. 1).

Logistical concerns including access to animals, producer reluctance to allow sampling, feasibility of sampling, and proper transport of samples from origin to sequencing laboratory all challenge the goal of obtaining a broad representation of cattle breeds and related species. We anticipate that not all participants in the project will have access to adequate laboratory facilities or to the pedigrees of sampled individuals in their herds. The BPC acknowledges that flexibility to accommodate disparate biological samples is required to achieve the goal of representation. We also welcome interested partners with shared or overlapping goals that already have invested resources, expertise, and/or animal samples. Consortium resources for generating bovine genome assemblies are finite, so samples must be prioritized based on the likelihood of quality outcomes from their use. To this end, the BPC proposes several quality standards for samples and materials that will be used to prioritize projects (see the “ Implementation” section).

The immediate goal of the pangenome project is the collection of assemblies for as many cattle breeds and related species as possible. This approach enables the immediate use of reference-quality assemblies for specific species/breeds using existing linear genome tools in the interim and allows for future assessment of data completeness before assembling a comprehensive pangenome resource. Eventually, the goal of the BPC is to construct a community-approved pangenome to be used for genomics in cattle, providing connections between studies while enhancing each study with an appropriate breed-specific context (Table 1).

**Table 1:** Goals of the BPC

• Establish collaborations with internal and external participants to facilitate genome assembly projects on diverse bovine species/breeds
• Coordinate bovine genome assembly efforts by publishing lists of on-going projects and by providing expertise to collaborating groups
• Mediate future bovine genome assembly efforts by providing a forum for the community to discuss issues, project ideas and limitations
• Provide updated recommendations and resources to the community to identify the best current resources to reduce losses in inter-study comparability resulting from use of different reference genome assemblies

## Role of the BPC

**Fig. 2:**
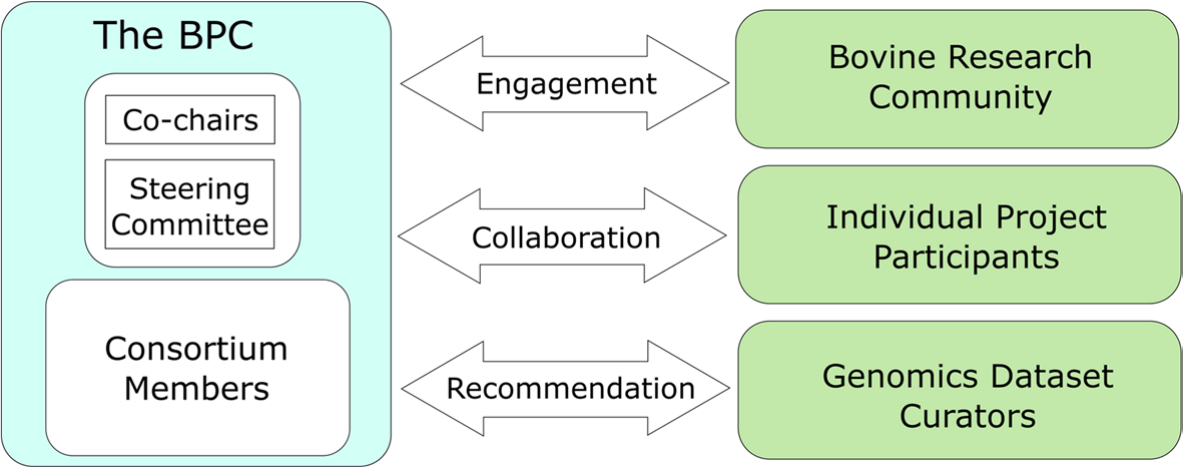
BPC cooperator outreach and engagement

The generation of reference-quality genome assemblies was enormously expensive and required extensive, specialized expertise as recently as 10 years ago [24]. Advances in both sequencing technologies and the algorithms for producing assemblies have vastly improved in the interim, reducing both cost and required expertise. The original conception of the BPC arose from the realization that entities worldwide would increasingly have the ability to generate such assemblies with no need for expert consultation. Uncoordinated efforts could therefore result in a squandering of the relatively meager resources available to the global livestock research community through unnecessary duplication of efforts and a rush to be the first to publish assemblies for a few high-impact breeds. The major benefits that the BPC can provide to the international community of cattle genomics researchers are (1) to facilitate collaborations for bovine genome assembly projects, (2) to establish a list of breeds/species assembly projects to assist in allocating resources, (3) to provide a forum for the discussion of the completion state of individual assembly projects, and (4) to create an organization to focus efforts on the development of a community-approved pangenome representation from the data. The BPC is organized into three distinct branches that will cooperate to reach a consensus on key policy and technical issues (Fig. 2). The BPC co-chairs will manage consortium resources and will consult with the steering committee to ensure that consortium objectives will be met. The larger BPC consortium will comprise all interested members of the research community that plan to directly work on the goals of the consortium. The entirety of the BPC will engage with the larger research community, genomics resource staff, and collaborating institutes to achieve consortium goals, while respecting ethical limits placed on data access.

The concept behind and intent of the BPC are to increase inclusivity and lower barriers to participation. The organizers are sensitive to concerns about the historical incidence of resource-rich entities benefiting at the expense of researchers and organizations with access to domestic species and breeds that would enhance the pangenome project. We are committed to avoiding the appearance or actuality of this problem, while also reaching out to promote the inclusion of historically under-represented geographic areas. Sequencing resources and computational infrastructure are available through the US Department of Agriculture to support interested researchers in these communities through this effort specifically. Although the USA and some other members’ countries are not signatories to the Nagoya Protocol (https://www.cbd.int/abs/text/), the BPC will adhere to the spirit of the access, benefit sharing, and compliance obligations of the protocol (https://www.cbd.int/abs/about/). The intent of the BPC is threefold: to facilitate bovine research in under-represented research communities, to support research programs through the judicious allocation of consortium resources, and to advise collaborators on the best practices for genome assembly and subsequent data analysis. We emphatically do not intend to “take over” any project, nor will we insist on any particular position in an authorship list. All data and analysis produced by the BPC will be considered the property of the sample provider, with full access and availability throughout the life of the project. The details of each collaboration will depend highly on the desires and situation of participants; however, the BPC’s driving goal is to facilitate the successful outcomes of a project to benefit the sample provider and the cattle research community as a whole. The only requirements are that the data is intended to be public within a year’s time of the assembly and that the assembly can be included in a public pangenome resource. The pangenome publication(s) resulting would acknowledge each constituent assembly and include authorship for participants if they desire. There is no intent to interfere with or prevent publication of individual genome assemblies or analysis, or pangenome constructions/representations with subsets of breeds. Rather, the ultimate goal is for the members of the BPC to lead the creation of a definitive community-standard representation that can be the basis of inter-study comparisons. Since we mandate that BPC participants keep direct ownership over the direction of their projects, they are able to pursue the specialized research into domestic or heritage breeds that they desire while still making substantial contributions to global cattle genome research efforts.

## Implementation

We recognize that public accessibility to component genome assemblies and user-friendly interfaces for making use of pangenome representation(s) in genomic studies are critically important to the success of the effort. We propose to join ongoing efforts at Ensembl and NCBI or initiate new ones as needed, in close concert with those data repositories to ensure open access and facilitate broad utility, and to provide a means for improving uniformity among disparate assembly efforts. We also plan to reach out to the FAANG initiative to enable sharing of resources that could be useful in the quality assessment of genome content [9, 25]. The creation, presentation, and application of pangenome representations of species genomes is an area of active investigation, and best practices are not yet fully agreed upon. New standards including, for example, the rGFA format for representing a reference pangenome graph [26], will be incorporated as they emerge and are refined. However, all emerging methods for representing pangenomes depend on sequence data and assemblies for a broad representation of the species, and thus, there is no advantage in waiting until a consensus is achieved to begin the collection of the underpinning data. One major consideration is that the input tissue type or DNA extraction method greatly influences the resulting quality of the DNA sequence from a sample. We recognize that it may not always be possible to obtain ideal samples for a breed, and there will be no absolute requirements for samples to meet all of our listed criteria provided that there are means to legally and ethically source them. The ideal sample is one that (1) is amenable to DNA extraction for long-read sequencing (i.e., not liver, hair roots, highly cartilaginous tissues, or tissues with high-fat content), (2) is free of concerns about pathogenic infections that prevent shipping to a sequencing laboratory (e.g., foot and mouth disease (FMD) virus, tuberculosis, or babesia), and (3) is large enough to support extraction of sufficient DNA for “greedy” long-read sequencing platforms/procedures. The use of long-read technologies vastly improves the contiguity, accuracy, and utility of de novo assemblies [17, 27] for the construction of pangenome representations. The latest assembly methods produce haplotype-resolved assemblies of two breeds from a single sample at a marginal increase in cost and reduced effort through the use of F1 animals produced by crossing breeds [4, 28]. These methods produce the most contiguous and accurate assemblies; therefore, a goal of the pangenome project includes a collection of F1 crosses between breeds where possible. Later-generation crossbred individuals will not be targeted for inclusion so long as the progenitor breeds are represented in the pangenome. Where the progenitors are not able to be sampled, crossbreds may be an appropriate way to capture missing diversity.

Pangenome approaches that use reference-guided assembly with either short or long reads have been proposed or performed, with the benefit that the cost is lower than complete de novo assembly and thus more animals can be processed for a given cost. A related approach is to assemble reads that fail to map to the genome with the supposition that resulting contigs would represent unique genomic sequences. The initial results indicate that a range of 3 to 10 megabases (Mb) of genome sequence that does not match the Hereford reference genome can be identified by sequencing animals of other breeds [20, 29]. This result is largely consistent with observations in human pangenome research, where anywhere from 5 to 300 Mb of non-reference sequence has been identified depending on the divergence between lineages. These methods are complementary to the proposed BPC approach of high-quality de novo assembly of one or a few haplotypes of a breed for as many breeds as possible and will serve to enhance the accuracy and representation of the final pangenome. The BPC will work to determine appropriate standards for consideration of an assembly for a particular breed, while noting that initial investigations of pangenome graphs indicate resilience in the face of lower-quality assemblies and low sensitivity to the inclusion of assemblies produced from very different methods and sequencing platforms [30]. This observation is promising since the evolution of technologies can be expected over the several years lifetime of the project; therefore, our present reliance on existing technologies should not prevent the utility of these assemblies when improvements come along (Table 2).

**Table 2:** The role of the BPC in developing the next generation of bovine genomics resources

• Generation of high-quality, haplotype-resolved reference genome assemblies for under-represented or highly divergent bovine breeds/species
• Consolidate the latest in bovine genome assemblies for use in pangenome resource construction
• Conduct a comparative analysis of bovine species to annotate conserved genomic regions that may correspond to species- or breed-specific phenotypic variation
• Organize experts and support the creation of graph-based pangenome reference(s) for cattle breeds to enable more accurate sequence-based genotyping

The cattle genome is roughly equivalent to the human genome in terms of the proportion of repetitive sequences, and the number of protein-coding genes and transcript isoforms per protein-coding gene is also similar [17]. We therefore anticipate that methods for pangenome construction, presentation, and implementation can make use of advances from the HPRC. There are a variety of approaches currently described that have varying degrees of dependence on an initial single reference assembly. Reference-guided assembly approaches based on de novo assembled contigs aligned to the reference have been used to identify insertion-deletion structural variants to add paths to a pangenome graph [31]. Mapping of WGS reads to the reference, followed by assembly of non-mapping reads assumed to represent the missing sequence in the reference, has also been applied [32, 33]. The most robust pangenome representations have resulted from independent de novo assembly and whole-genome alignment [26, 34] as demonstrated by a comparison of approaches to the same dataset [35].

Effective means for interrogating pangenomes that are computationally tractable for further variant discovery based on mapping of inexpensive short-read data at the population scale are being developed [36–39]. Bovine pangenomes with limited numbers of breeds have been reported [19, 20, 29] that used combined methods of aligned assemblies with the addition of structural variants identified by mapping of short reads to the graph created from those aligned assemblies. These efforts underscore the need for the research community to come to an agreement on a single representation that is robust for multiple comparative analyses. Such a reference will need to accommodate short read-based variation and genotyping studies employed in cattle genomics projects, to provide a common coordinate system for comparison across studies and serve as a foundation for annotation of functional genome features. As this reference will be used as a basis for genetic sequence comparison in research and industry, concrete thresholds for a public release must be identified by the community (with mediation by the BPC) to avoid frequent iterations of resources that disrupt ongoing work. We do not anticipate that any pangenome graph prepared will be the final product as future work may produce ever more effective means to represent pangenomes or further iteration may be necessary to represent cryptic DNA sequences missed in our original survey. However, just as the original UMD3.1 cattle genome assembly [40] was useful for cross-study comparisons for over a decade, we believe that a useful, community-approved pangenome reference can provide a stable means for similar time frames.

Genome assembly efforts such as the Vertebrate Genomes Project [41] have emphasized the use of a common technology and methodology for the production of assemblies to provide uniformity and prevent biases when comparing genome architecture across species. An evaluation of pangenome construction in cattle suggested that a bovine pangenome can make use of genome assemblies of breeds at variable levels of quality, input data type, or assembly method [30]. Best practices for aligning genomes, presenting pangenome graphs, and performing variant detection on those graphs are rapidly evolving and are likely to continue to change in the foreseeable future. However, the observation that the pangenome will be robust to variability in assembly parameters supports our contention that the community can go forward with generating genome assemblies for global bovine species as soon as possible. This makes the most important constraint the availability of high-quality samples from diverse bovine species/breeds for genome assembly. Since the collection of samples, generation of sequence data, and assembly of many hundreds of breeds is unlikely to be completed in the next year or two, it is likely that by the time we have the assemblies in hand, the best approach or at least useful non-ideal approach will be generally agreed upon for generating the community standard bovine pangenome. We suggest that until such time, mapping to the Hereford ARS-UCD1.2 reference assembly for cross-study comparisons will continue to be the most useful approach rather than study-specific pangenome graphs for variant discovery. The use of such intermediate resources poses the risk of immediately dating variant calling results while the community coalesces on a final representative genomics resource. At that time, the BPC will plan to provide leadership in the selection of pangenome resources suitable for different applications by using community-driven feedback on developed resources. By serving as an intermediary and facilitator for the recommendation of the final pangenome resource, the BPC plans to reduce confusion and improve cross-study comparability for future bovine resequencing projects.

## Conclusion

The BPC was established as a means to coordinate bovine global genome assembly efforts and not as a means to override, interfere, or take credit for local efforts. We encourage researchers that want to participate in the pangenome effort to join us. The initial assembly of diverse bovine species/breeds is supported by the US Department of Agriculture in part because of the historical role of the Agricultural Research Service (ARS) in the early cattle genome assembly efforts, development of methods for genome assembly, and ongoing presence in the generation of bovine genome assemblies. However, the BPC has an international steering committee and encourages open discussion of any topics related to data sharing, publication strategy, or logistics of sampling animals around the world. Resources for creating genome assemblies are available through ARS, and we are open to discussion of providing sequencing and/or assembly for breeds where local resources are not available. Any such collaborations will provide only the support requested, i.e., any combination of sequencing, assembly, data deposition, or advice. Experts participating in the consortium can assist in manuscript writing as requested. The sole requirement is that ARS-funded genome assemblies will be made public and will be available for the final pangenome representation. All collaborators will be offered the opportunity for coauthorship on manuscript(s) describing the community-standard pangenome, assuming they materially contributed to composite assemblies.

A list of breeds that have been assembled or have allocated resources to support assembly will be maintained at https://bovinepangenome.github.io/. The BPC will continue with the active outreach that we have been engaged in during the pandemic, but welcome contact from anyone that has access to cattle breeds or bovine species not currently on our list, or for which there is evidence that sufficient within-breed or within-species diversity exists to make multiple assemblies worthwhile.

## Supplementary Information

**Additional file 1.**
