# Supplementary material for: The Bovine Pangenome Consortium: democratizing production and accessibility of genome assemblies for global cattle breeds and other bovine species
Source: Genome Biol. 2023 Jun 19;24:139. doi: 10.1186/s13059-023-02975-0 (PMC10278262; doi:10.1186/s13059-023-02975-0)
Supplement: Supplementary file 1 — Additional file 1. [file 13059_2023_2975_MOESM1_ESM.docx]

**1^st^ round**

**Reviewer 1**

The Bovine Pangenome Consortium (BPC) aims to develop a more complete representation of cattle genomic diversity by assembling the pangenome covering world-wide cattle breeds and other Bovidae species. The cattle pangenome will overcome several limitations inherent in studies using singular, linear genome assemblies:

1)The first is to identify structural variants which are particularly difficult to accurately characterize using short reads or even long reads mapping to a single-haplotpe reference assembly.

2)The second is to detect insertions which are particularly recalcitrant to identify without orthogonal genomic information as sequence reads from haplotpes containing the insertion fail to properly ma to the reference.

3)The third is to rectify the incorrect mapping of sequence reads with low similarity to the reference assembly due to population variation in genome sequence.

The effort of BPC will promote the construction of a community-agreed, mutually beneficial pangenome representation to fuel the discovery of genetic markers for precision breeding for important traits.

Generally speaking, the description of the goals and the role of BPC is concise and clear. I recommend acceptance of the manuscript after minor revision.

Minor Issues:

1.In the Sample Collection part of Figure 2, the authors indicated the collection of one reference individual or trio for sequencing. But structural variations and insertions do exist within one cattle breed population., sampling one individual or the trio may not be enough to capture all variations of the investigated breed. How many individuals of one breed are sufficient to represent the investigated cattle breed?

2.Some cattle breeds are cultivated from the hybridization between Bos taurus taurus and Bos taurus indicus, are they suitable to be included in pangenome construction?

**Reviewer 2**

The manuscript entitled "The Bovine Pangenome Consortium: democratizing production and accessibility of genome assemblies for global cattle breeds and other bovine species" has been submitted to Genome Biology for publication as a perspective/review-type article. The manuscript details how a systematic pangenome approach to cataloguing and representing the full spectrum of Bovini genomic diversity will significantly enhance basic and applied research in domestic cattle and related species. Because the manuscript is intended to be a perspective/review article, some of the checkbox/pulldown menu questions in the online Review system do not apply and I have answered 'Yes' to these so that this Reviewer evaluation can be submitted (e.g., "Is the study design appropriate to answer the research question [including the use of appropriate controls], and are the conclusions supported by the evidence presented?" or "Are the methods sufficiently described to allow the study to be repeated?"). In addition, the suggested structure of the Reviewer Comments to Author text is not appropriate and I have structured my evaluation in a bulleted form that is more appropriate for a perspective/review article.

* OVERVIEW: The manuscript is, for the most part, well written and provides a very good overview of the Bovine Pangenome Consortium (BPC) and their plans for generating graph genome-based pangenomes for basic and applied research applications in cattle and related species, which represent some of the most important domestic animals used for meat, milk, hides, traction, and manure across the globe. It is important to emphasise that the outputs of the BPC can be directly and rapidly applied in genome-enabled livestock breeding programmes that will be critically important to 21st century food security and mitigating the environmental impact of animal agriculture. I have bulleted some recommended and suggested edits/changes to the manuscript below. When these are incorporated into a revised manuscript, I would consider it suitable for publication in Genome Biology.

* RELATIONSHIP TO THE FUNCTIONAL ANNOTATION OF ANIMAL GENOMES (FAANG) INITIATIVE: It is important to keep in mind that Genome Biology has a general readership encompassing researchers working with a variety of animal, plant, and microbial species and across a wide range of scientific disciplines that leverage genomic technologies, methodologies and computational approaches. Therefore, most of the readership will not be familiar with many of the terms, concepts, and abbreviations used routinely by animal geneticists and livestock genome biologists. For example, the Functional Annotation of Animal Genomes (FAANG) Initiative (www.faang.org) will be key to both interpreting and exploiting the scientific outputs from the BPC and I would strongly recommend that the cattle FAANG project is explained in more detail in the article. At present, the FAANG Initiative is briefly mentioned in passing on lines 86 and 268 and most of the Genome Biology readership may only know "FAANG" as the Facebook, Amazon, Apple, Netflix, and Google group of tech stocks! A brief overview of the rationale and aims of the cattle FAANG project and how it is inspired by similar projects in human (i.e., ENCODE, Roadmap Epigenomics Consortium) and mouse (i.e., Mouse ENCODE) would be useful. A new Box 1 might be good for this.

* GENOME-ENABLED BREEDING: MARKER-ASSISTED SELECTION VERSUS GENOMIC SELECTION: The section from line 80 to line 99 is quite confusing and unclear in how it describes the application of genomic information to cattle breeding programmes and genetic improvement. I would recommend that the authors rewrite this section, clearly distinguishing between marker-assisted selection (MAS) for managing monogenic (e.g., double muscling and the MSTN gene) or oligogenic (e.g., coat colour) traits versus genome-wide selection approaches for polygenic traits (genomic selection), which is based on phenotyping and genotyping reference/training populations and estimation of genomic estimated breeding values (GEBVs) for animals that have genome-wide genotype data but that do not have linked phenotype data. Again, it is important to keep in mind the general readership of Genome Biology and it might be useful to make the connection between genomic prediction in livestock and polygenic risk scores for complex traits in human populations (see Wray et al. 2019: https://doi.org/10.1534/genetics.119.301859). It may also be useful to briefly mention how biology-driven genomic prediction and selection is emerging as functional genomics information (from FAANG-orientated research) is being incorporated into statistical methodologies for animal breeding.

* RELATIONSHIP BETWEEN THE BPC WEBSITE AND THE ARTICLE: I think that the current (and possibly the permanent and official) website for the Bovine Pan-Genome Consortium (https://bovinepangenome.github.io) should be introduced earlier in the manuscript, not in the last paragraph of the Conclusion section. The authors may also want to consider including the BPC web address in the Abstract so it is immediately accessible in the scientific literature databases (PubMed, WoS, Scopus etc.). If a different URL is planned or about to be implemented (e.g., www.bovinepangenome.org) then this should be the URL used in the article. I also note that the website uses the hyphenated form of "pan-genome", whereas the manuscript uses the shorter and much more widely used "pangenome" form.

* THE SOCIETAL AND ETHICAL ASPECTS OF THE BPC: For the most part, the authors do a good job of explaining how the BPC will be inclusive and encourage the participation of researchers from under-resourced and underrepresented communities (lines 234-260). Presumably many of these researchers will be in developing countries and the Global South where much of cattle (and related species) genomic diversity resides. It is therefore surprising that no mention is made of the Nagoya Protocol on Access and Benefit Sharing (ABS), which applies to researchers who wish to access livestock genetic resources (including biological material containing DNA). With some notable exceptions such as the United States, Canada, and New Zealand, most countries have signed and ratified the Protocol (www.cbd.int/abs/nagoya-protocol/signatories). Most of the Bovini genome sequences used for BPC research efforts have been, and will be, generated from animals native to these countries. Therefore, I would strongly recommend that the authors include a statement about the Nagoya Protocol and indicate that BPC will adhere to the access, benefit-sharing, and compliance obligations of the Protocol (https://www.cbd.int/abs/about). In the long run, this could help ward off potential misunderstanding, controversy, and negative publicity about the aims and goals of the BPC.

* FIGURES 1 AND 2: In their present form, Figures 1 and 2 are quite crude and not particularly informative, especially Figure 2. I would strongly recommend that Figure 2is re-imagined and re-drawn using Figure 1 in Wang et al. (2022: https://doi.org/10.1038/s41586-022-04601-8) for inspiration. In particular, it would be good to include a phylogenetic/graphical representation of the key Bovini species that will be part of the BPC research programme and it would also be useful to incorporate a simple graphical representation of the bovine pangenome concept. It might also be another point in the article to emphasize the importance of the Nagoya Protocol and the relationship between the "The BPC" and "The Cooperator" etc.

* MINOR EDITS/CHANGES:

o The citation markers should be reformatted to the standard Genome Biology format (numbered in square brackets).

o Line 60: "to avoid the pilfering sensitive information" is unusual phraseology - why not just "to ensure the security of sensitive information"? Also, "the exclusion of international groups" is very vague - can the meaning here be clarified in the revised manuscript?

o Lines 100-105: Taurine cattle ("Bos taurus") are mentioned here but there is no mention of "Bos indicus" (zebu) cattle and the myriad hybrid populations that exist between the two subspecies. For understanding the importance and utility of the pangenome approach, I think it would be useful to introduce and describe zebu cattle and taurine/indicine hybrid populations here, also indicating that although evolutionary distinct, the two subspecies are completely interfertile and that admixed populations are widespread in Africa, South America, Southeast Asia and elsewhere. Also, line 105, "natural constraints on mating" is strange phraseology and needs to be clarified. See also the further points about making the taxonomic identifiers consistent.

o Line 143: "Sapiens" should be "sapiens".

o Line 146: the taxonomy is not consistent with previous sections (see lines 100-105): "Bos taurus taurus" and "Bos taurus indicus" are the verified Integrated Taxonomic Information System (ITIS: www.itis.gov) recommended identifiers for the two subspecies; however, the last word should also be italicised. In the earlier section, it might make sense to introduce the two subspecies as "Bos taurus taurus" and "Bos taurus indicus" but then indicate that the shorter binomial "species" forms ("Bos taurus" and "Bos indicus") will be used for simplicity.

o Lines 157-159: the taxonomic species identifiers should also be included, not just the common names. Again, the ITIS database can be consulted for these. In addition, this is where a relatively simple phylogeny/evolutionary tree representation would be useful (perhaps incorporated into a revised version of Figure 1).

o Line 263: "facile" is an unusual word choice here, particularly because it has negative connotations and will have non-native English speakers scrambling for their dictionaries. Why not just "user-friendly and intuitive"?

**Authors’ response**

Reviewer #1: The Bovine Pangenome Consortium (BPC) aims to develop a more complete representation of cattle genomic diversity by assembling the pangenome covering world-wide cattle breeds and other Bovidae species. The cattle pangenome will overcome several limitations inherent in studies using singular, linear genome assemblies:

1)The first is to identify structural variants which are particularly difficult to accurately characterize using short reads or even long reads mapping to a single-haplotpe reference assembly.

2)The second is to detect insertions which are particularly recalcitrant to identify without orthogonal genomic information as sequence reads from haplotpes containing the insertion fail to properly ma to the reference.

3)The third is to rectify the incorrect mapping of sequence reads with low similarity to the reference assembly due to population variation in genome sequence.

The effort of BPC will promote the construction of a community-agreed, mutually beneficial pangenome representation to fuel the discovery of genetic markers for precision breeding for important traits.

Generally speaking, the description of the goals and the role of BPC is concise and clear. I recommend acceptance of the manuscript after minor revision.

Minor Issues:

1.In the Sample Collection part of Figure 2, the authors indicated the collection of one reference individual or trio for sequencing. But structural variations and insertions do exist within one cattle breed population., sampling one individual or the trio may not be enough to capture all variations of the investigated breed. How many individuals of one breed are sufficient to represent the investigated cattle breed?

We agree that this is an important question to investigate and have added some additional text to further drive the point (lines 155-159 & 370-374). Unfortunately, we have no answer at the moment.

“Our ability to represent all structural variation will be a function of allele frequencies and the partitioning of variation between breeds. It will therefore be important to test the necessity of including multiple assemblies within more cosmopolitan breeds where animal numbers are high, or the breed may have had its genome shaped by different selection pressures.”

“The BPC will continue with the active outreach that we have been engaged in during the pandemic, but welcome contact from anyone that has access to cattle breeds or bovine species not currently on our list, or for which there is evidence that sufficient within-breed or within-species diversity exists to make multiple assemblies worthwhile.”

2.Some cattle breeds are cultivated from the hybridization between Bos taurus taurus and Bos taurus indicus, are they suitable to be included in pangenome construction?

Thanks for the question, we have tried to clarify this (lines 272-275).

“Later generation crossbred individuals will not be targeted for inclusion so long as the progenitor breeds are represented in the pangenome. Where the progenitors are not able to be sampled, crossbreds may be an appropriate way to capture missing diversity.”

Reviewer #2: The manuscript entitled "The Bovine Pangenome Consortium: democratizing production and accessibility of genome assemblies for global cattle breeds and other bovine species" has been submitted to Genome Biology for publication as a perspective/review-type article. The manuscript details how a systematic pangenome approach to cataloguing and representing the full spectrum of Bovini genomic diversity will significantly enhance basic and applied research in domestic cattle and related species. Because the manuscript is intended to be a perspective/review article, some of the checkbox/pulldown menu questions in the online Review system do not apply and I have answered 'Yes' to these so that this Reviewer evaluation can be submitted (e.g., "Is the study design appropriate to answer the research question [including the use of appropriate controls], and are the conclusions supported by the evidence presented?" or "Are the methods sufficiently described to allow the study to be repeated?"). In addition, the suggested structure of the Reviewer Comments to Author text is not appropriate and I have structured my evaluation in a bulleted form that is more appropriate for a perspective/review article.

* OVERVIEW: The manuscript is, for the most part, well written and provides a very good overview of the Bovine Pangenome Consortium (BPC) and their plans for generating graph genome-based pangenomes for basic and applied research applications in cattle and related species, which represent some of the most important domestic animals used for meat, milk, hides, traction, and manure across the globe. It is important to emphasise that the outputs of the BPC can be directly and rapidly applied in genome-enabled livestock breeding programmes that will be critically important to 21st century food security and mitigating the environmental impact of animal agriculture. I have bulleted some recommended and suggested edits/changes to the manuscript below. When these are incorporated into a revised manuscript, I would consider it suitable for publication in Genome Biology.

* RELATIONSHIP TO THE FUNCTIONAL ANNOTATION OF ANIMAL GENOMES (FAANG) INITIATIVE: It is important to keep in mind that Genome Biology has a general readership encompassing researchers working with a variety of animal, plant, and microbial species and across a wide range of scientific disciplines that leverage genomic technologies, methodologies and computational approaches. Therefore, most of the readership will not be familiar with many of the terms, concepts, and abbreviations used routinely by animal geneticists and livestock genome biologists. For example, the Functional Annotation of Animal Genomes (FAANG) Initiative (<https://www.faang.org/>) will be key to both interpreting and exploiting the scientific outputs from the BPC and I would strongly recommend that the cattle FAANG project is explained in more detail in the article. At present, the FAANG Initiative is briefly mentioned in passing on lines 86 and 268 and most of the Genome Biology readership may only know "FAANG" as the Facebook, Amazon, Apple, Netflix, and Google group of tech stocks! A brief overview of the rationale and aims of the cattle FAANG project and how it is inspired by similar projects in human (i.e., ENCODE, Roadmap Epigenomics Consortium) and mouse (i.e., Mouse ENCODE) would be useful. A new Box 1 might be good for this.

We agree with the reviewer that that the FAANG consortium should be mentioned as it is a potential cooperator and beneficiary of the data that will be produced from the BPC. However, we believe that a discursive description of the FAANG consortium would detract from the overall description of the BPC in this white paper. The BPC was founded separately from the FAANG consortium, so a lengthy description of the goals of FAANG would provide little benefit to our white paper text, while potentially confusing the reader as to the organization of both groups (lines 79-81).

“…and the establishment of the international Functional Annotation of Animal Genomes “FAANG to Fork” initiative [9], a project analogous to the human ENCODE project [10].”

* GENOME-ENABLED BREEDING: MARKER-ASSISTED SELECTION VERSUS GENOMIC SELECTION: The section from line 80 to line 99 is quite confusing and unclear in how it describes the application of genomic information to cattle breeding programmes and genetic improvement. I would recommend that the authors rewrite this section, clearly distinguishing between marker-assisted selection (MAS) for managing monogenic (e.g., double muscling and the MSTN gene) or oligogenic (e.g., coat colour) traits versus genome-wide selection approaches for polygenic traits (genomic selection), which is based on phenotyping and genotyping reference/training populations and estimation of genomic estimated breeding values (GEBVs) for animals that have genome-wide genotype data but that do not have linked phenotype data. Again, it is important to keep in mind the general readership of Genome Biology and it might be useful to make the connection between genomic prediction in livestock and polygenic risk scores for complex traits in human populations (see Wray et al. 2019: <https://academic.oup.com/genetics/article/211/4/1131/5931508> ). It may also be useful to briefly mention how biology-driven genomic prediction and selection is emerging as functional genomics information (from FAANG-orientated research) is being incorporated into statistical methodologies for animal breeding.

We agree that the wording of this section was confusing, so we have refined the text to use some of the terms suggested by the reviewer. We have made some connections between the efficiency of genomic selection and CADD scores to highlight the importance of genotype-to-phenotype prediction as it would relate to animal breeding. While there is much detail we could provide here on each subject individually, we have opted for a concise summary in order to focus more on the rationale for and approach of the BPC. Our new additions include the following text (lines 81-83 and 85-90)

“Prior work in the development of predictive models for complex trait phenotypes from a fixed set of genetic markers, termed Genomic Selection, has resulted in substantial benefit for animal breeders [11,12].”

“However, the accuracy of genomic selection on certain phenotypic traits is greatly impacted by environmental effects and the influence of high effect, low-frequency genetic variants [citation]. Improved reference genome assemblies and efforts to predict combined annotation-dependent depletion scores (CADD; [15]) for all bases in the genome of a species could allow for the inclusion of high-effect, low-frequency variants into generalized genomic selection models.”

* RELATIONSHIP BETWEEN THE BPC WEBSITE AND THE ARTICLE: I think that the current (and possibly the permanent and official) website for the Bovine Pan-Genome Consortium (<https://bovinepangenome.github.io/>) should be introduced earlier in the manuscript, not in the last paragraph of the Conclusion section. The authors may also want to consider including the BPC web address in the Abstract so it is immediately accessible in the scientific literature databases (PubMed, WoS, Scopus etc.). If a different URL is planned or about to be implemented (e.g., [www.bovinepangenome.org](http://www.bovinepangenome.org)) then this should be the URL used in the article. I also note that the website uses the hyphenated form of "pan-genome", whereas the manuscript uses the shorter and much more widely used "pangenome" form.

We agree with the reviewer and have added the URL to the abstract. We have also more generally introduced the BPC earlier in the manuscript (lines 70-73). The text of the website has also been updated.

“Because no similar singular funding source is available for agricultural genomics, the Bovine Pangenome Consortium (BPC) has been launched (https://bovinepangenome.github.io/) to coordinate distributed efforts within the global bovine genomics community towards achieving similar ends.”

* THE SOCIETAL AND ETHICAL ASPECTS OF THE BPC: For the most part, the authors do a good job of explaining how the BPC will be inclusive and encourage the participation of researchers from under-resourced and underrepresented communities (lines 234-260). Presumably many of these researchers will be in developing countries and the Global South where much of cattle (and related species) genomic diversity resides. It is therefore surprising that no mention is made of the Nagoya Protocol on Access and Benefit Sharing (ABS), which applies to researchers who wish to access livestock genetic resources (including biological material containing DNA). With some notable exceptions such as the United States, Canada, and New Zealand, most countries have signed and ratified the Protocol (https://www.cbd.int/abs/nagoya-protocol/signatories/) . Most of the Bovini genome sequences used for BPC research efforts have been, and will be, generated from animals native to these countries. Therefore, I would strongly recommend that the authors include a statement about the Nagoya Protocol and indicate that BPC will adhere to the access, benefit-sharing, and compliance obligations of the Protocol (<https://www.cbd.int/abs/about/>). In the long run, this could help ward off potential misunderstanding, controversy, and negative publicity about the aims and goals of the BPC.

This is a good point. It wasn’t originally included because, as you mentioned, the US is not a signatory to the Protocol but stating that we will adhere to the spirit of the Protocol will hopefully help lessen concern (lines220-223).

“Although the United States and some other member's countries are not signatories to the Nagoya Protocol (https://www.cbd.int/abs/text/), the BPC will adhere to the spirit of the access, benefit-sharing, and compliance obligations of the Protocol (https://www.cbd.int/abs/about/).”

* FIGURES 1 AND 2: In their present form, Figures 1 and 2 are quite crude and not particularly informative, especially Figure 2. I would strongly recommend that Figure 2is re-imagined and re-drawn using Figure 1 in Wang et al. (2022: <https://www.nature.com/articles/s41586-022-04601-8>) for inspiration. In particular, it would be good to include a phylogenetic/graphical representation of the key Bovini species that will be part of the BPC research programme and it would also be useful to incorporate a simple graphical representation of the bovine pangenome concept. It might also be another point in the article to emphasize the importance of the Nagoya Protocol and the relationship between the "The BPC" and "The Cooperator" etc.

As per the reviewer’s suggestion, we have removed figure 2. In this revision, we have added a graphical representation of a Bovini species pangenome as suggested as Figure 1with a phylogenetic representation of the key Bovini species that will be part of the BPC project. We note that Bovini species inclusion is subject to change based on the participation of external groups in the BPC; however, we agree with the reviewer that such a graphic would be helpful to potential participants in this project.

“Figure 1: Bovine species relationships and pangenome conceptualization. Single nucleotide and structural variation between species/subspecies/breeds are depicted as different paths through a multi-species pangenome.”

* MINOR EDITS/CHANGES:

o The citation markers should be reformatted to the standard Genome Biology format (numbered in square brackets).

done

o Line 60: "to avoid the pilfering sensitive information" is unusual phraseology - why not just "to ensure the security of sensitive information"? Also, "the exclusion of international groups" is very vague - can the meaning here be clarified in the revised manuscript?

We have reworded this section (lines 66-67).

“The charter for this effort includes the ethical access of samples to avoid the exploitation of local populations and outreach to international groups as explicit goals”

o Lines 100-105: Taurine cattle ("Bos taurus") are mentioned here but there is no mention of "Bos indicus" (zebu) cattle and the myriad hybrid populations that exist between the two subspecies. For understanding the importance and utility of the pangenome approach, I think it would be useful to introduce and describe zebu cattle and taurine/indicine hybrid populations here, also indicating that although evolutionary distinct, the two subspecies are completely interfertile and that admixed populations are widespread in Africa, South America, Southeast Asia and elsewhere. Also, line 105, "natural constraints on mating" is strange phraseology and needs to be clarified. See also the further points about making the taxonomic identifiers consistent.

We have added mention of Bos indicus (lines 104, 112) as well as the potential for inclusion of crossbred animals (lines 272-275)

“We suggest the development of reference-quality genome assemblies for as many of the existing distinct cattle breeds, including representatives of both subspecies, Bos taurus taurus and Bos taurus indicus, hensforth taurine and indicine, as practical to modernize the cattle reference genome.”

“Taurine and indicine cattle, riverine and swamp buffalo (Bubalus bubalis), yak (Bos grunniens), and gayal (Bos frontalis) have extensive histories of domestication which include the recent formation of breeds due to intensive selection for agricultural food products.”

“Later generation crossbred individuals will not be targeted for inclusion so long as the progenitor breeds are represented in the pangenome. Where the progenitors are not able to be sampled, crossbreds may be an appropriate way to capture missing diversity.”

o Line 143: "Sapiens" should be "sapiens".

corrected

o Line 146: the taxonomy is not consistent with previous sections (see lines 100-105): "Bos taurus taurus" and "Bos taurus indicus" are the verified Integrated Taxonomic Information System (ITIS: <https://www.itis.gov/>) recommended identifiers for the two subspecies; however, the last word should also be italicised. In the earlier section, it might make sense to introduce the two subspecies as "Bos taurus taurus" and "Bos taurus indicus" but then indicate that the shorter binomial "species" forms ("Bos taurus" and "Bos indicus") will be used for simplicity.

Corrected (line144)

“…population size of the global population to the extent observed for *Homo sapiens*”

o Lines 157-159: the taxonomic species identifiers should also be included, not just the common names. Again, the ITIS database can be consulted for these. In addition, this is where a relatively simple phylogeny/evolutionary tree representation would be useful (perhaps incorporated into a revised version of Figure 1).

Thanks for the suggestion, as indicated above, we have added this to the new Figure 1.

o Line 263: "facile" is an unusual word choice here, particularly because it has negative connotations and will have non-native English speakers scrambling for their dictionaries. Why not just "user-friendly and intuitive"?

Thanks for catching this, we agree (lines 244-246).

“We recognize that public accessibility to component genome assemblies and user-friendly interfaces for making use of pangenome representation(s) in genomic studies are critically important to success of the effort.”
